# Detection of toxoplasmosis in pets and stray cats through molecular and serological techniques in Khyber Pakhtunkhwa, Pakistan

**DOI:** 10.1186/s12917-021-03064-9

**Published:** 2021-11-19

**Authors:** Abdul Majid, Nisar Ahmad, Sumbal Haleem, Noor ul Akbar, Shehzad Zareen, Muhammad Taib, Sanaullah Khan, Rafiq Hussain, Sohail

**Affiliations:** 1 Department of Zoology, Kohat University of Science and Technology, Kohat, Pakistan; 2 Institute of Basic Sciences, Khyber Medical University, Peshawar, Pakistan; 3 Department of Zoology, University of Peshawar, Peshawar, Pakistan; 4 Department of Biological Sciences, University of Lakki, Marwat, KP Pakistan; 5 Institute of Biology/Plant Physiology, Humboldt-University Zü Berlin, 10115 Berlin, Germany

**Keywords:** *T. gondii*, Seroprevalence, Toxoplasmosis, Cats, Khyber Pakhtunkhwa, ELISA, PCR

## Abstract

**Background:**

*Toxoplasma gondii* is an important parasite that belongs to the phylum *Apicomplexa*, distributed globally, causing major health issues for a wide range of hosts, including humans, native and wild animals.

**Methods:**

In the present study, we detected IgG and IgM antibodies through an ELISA kit and DNA of *T. gondii* through PCR in 197 pets and stray cats in Peshawar, Charsadda, Mardan, and Kohat districts of Khyber Pakhtunkhwa (Pakistan) to estimate the existence of feline toxoplasmosis.

**Results:**

The current study revealed that stray cats have a significant infection rate of *T. gondii* (74.6%) as compared to pet cats (25.4%). In all the four districts, the prevalence of *T. gondii* was pointedly higher in district Kohat (95.5%) in the feline population. In comparison to the female (75.18%) and male (both pets and stray) cats have a maximum infection of (81.66%) non-significantly. The prevalence of *T. gondii* was observed to be significantly higher (91.66%) in the older and greater than 4 year old population of cats as compared to the younger ones. In poor health condition, the cat populations has a higher risk of infection of 92.3% as compared to healthy and poor body condition (73.91%) and (82.6%) respectively. The chronic and reactivated chronic conditions of toxoplasmosis were higher (58.37%) as compared to the acute condition.

**Conclusion:**

It has been concluded that toxoplasmosis is widely spread in the studied population.The outcomes of the present study show that *T. gondii* infection has a significant impact on the type of cat, age, and area, which implies a serious threat to human beings. Therefore, genotyping of *T. gondii* strains from different hosts is needed to forecast the current approach for prevention and control of this zoonotic parasite.

## Background

*Toxoplasma gondii* is an important zoonotic parasite of the phylum *Apicomplexa* causing toxoplasmosis globally [1]. Felines are the definitive hosts of *T. gondii*, whereas humans and all other warm-blooded animals are intermediate hosts [2]. In humans, the most common pathway of *T. gondii* transmission is through consumption of undercooked meat containing cysts, poorly washed vegetables, and water or soil polluted with oocysts [3]. Field workers are at high risk of *T. gondii* infection as felines are the only specie that excretes resistant oocysts into the environment [4]. Cats excrete approximately 20 million oocysts within 3 to 18 days of infection [5]. Toxoplasmosis is usually subclinical or asymptomatic, but sometimes it can cause fever, anorexia, lethargy, eye swelling, intestinal pain, and neurological disorders, or it may rarely kill small kittens by making lesions in their intestinal epithelial layer [6, 7].

Number of studies conducted on toxoplasmosis in the pet and stray cat population [8, 9] across the globe using diverse serological and molecular techniques. Epidemiology of toxoplasmosis in the pet and stray cat population of Pakistan has not been thoroughly investigated and little is known about the frequency and diversity of the disease (Table 1). The key determinant of the decision to undertake this study was the lack of data on toxoplasmosis in the Khyber Pakhtunkhwa pet and stray cat population. Our goal was to determine the prevalence of *T. gondii* in pets and stray cats using both serological (ELISA) and molecular methods (PCR).

**Table 1:** Summary of the prevalence and genotypes of *T. gondii* in cats in Pakistan

Article Title	District	Province	Prevalence	Stray/Pet	Testing Method	Genotype	Ref.
Sero-epidemiological and haematological studies on toxoplasmosis in cats, dogs and their owners in Lahore, Pakistan	Lahore	Punjab	56%	Both	LAT	N/A	[11]
Prevalence of *Toxoplasma gondii* oocysts through Copro-PCR in cats at Pet Center (UVAS), Lahore, Pakistan	Lahore	Punjab	2.3%	Pet	Copro-PCR	N/A	[22]
Serological survey of *Toxoplasma gondii* in dogs and cats	Faisalabad	Punjab	60%	Both	LAT	N/A	[12]
Sequence Analysis of SAG2 of Feline *Toxoplasma gondii* Oocysts in Pakistan	Lahore	Punjab	N/A	Pet	PCR	atypical strain	[23]
Seroprevalence of IgG and IgM antibodies and associated risk factors for toxoplasmosis in cats and dogs from subtropical arid parts of Pakistan	Rawalpindi, Jhelum, Chakwal, and Attock	Punjab	26.43%	Pet	ELISA	N/A	[24]

## Material and methods

### Study area, sample collection and procession

This cross-sectional study was conducted in the districts of Peshawar, Charsadda, Mardan, and Kohat, Khyber Pakhtunkhwa, Pakistan with geographical coordinates of 34.0151° N, 71.5249° E, 34.1682° N, 71.7504° E, 34.1989° N, 72.0231° E, and 33.5889° N, 71.4429° E respectively. Our study focused on both pet and stray cats of any sex, race and age above 4 months. The study was carried out in compliance with the ARRIVE guidelines (http://www.nc3rs.org.uk/page.asp?id=1357) as “All experiments were performed in accordance with relevant national/international/institutional guidelines and regulations”. All domesticated cats were considered pet cats, whereas all non-domesticated cats were considered stray cats in this study. A total of 197 blood samples were collected randomly from the cats of the study area, where 147 samples were taken from stray cats and 50 samples from pet cats. At the time of sampling from pet cats, each cat owner was briefed verbally about the current research project and an informed consent form was signed by each cat owner having complete written details of the current study. Blood samples from cats were collected with the help of a veterinarian. By using tranquilizer gun, cats were first anesthetized with Ketamine-acepromazine by shooting them into their neck/shoulder muscles. No animal was euthanized who were visibly ill/moribund in this study. Then, under aseptic conditions, through 3 ml BD syringes (Germany), by puncturing the femoral vein of each subject, 3 ml of blood was extracted. 2 ml of blood was directly moved for serological tests into gel and clot activator tubes, while 1 ml of blood was stored for molecular studies in the EDTA tube. Serum and blood samples were properly labelled and stored at -20 °C until further experimentation.

### Sero-diagnosis (Indirect ELISA)

For serodiagnosis, the indirect ELISA method was followed for the detection of IgG and IgM antibodies against *T.gondii*. The prescribed protocol of the kit was followed for the assay (Saronno VA Volonterio, 36a, 21,047, Italy).

### Molecular diagnosis

#### DNA extraction, amplification and electrophoresis

**Fig. 1:**
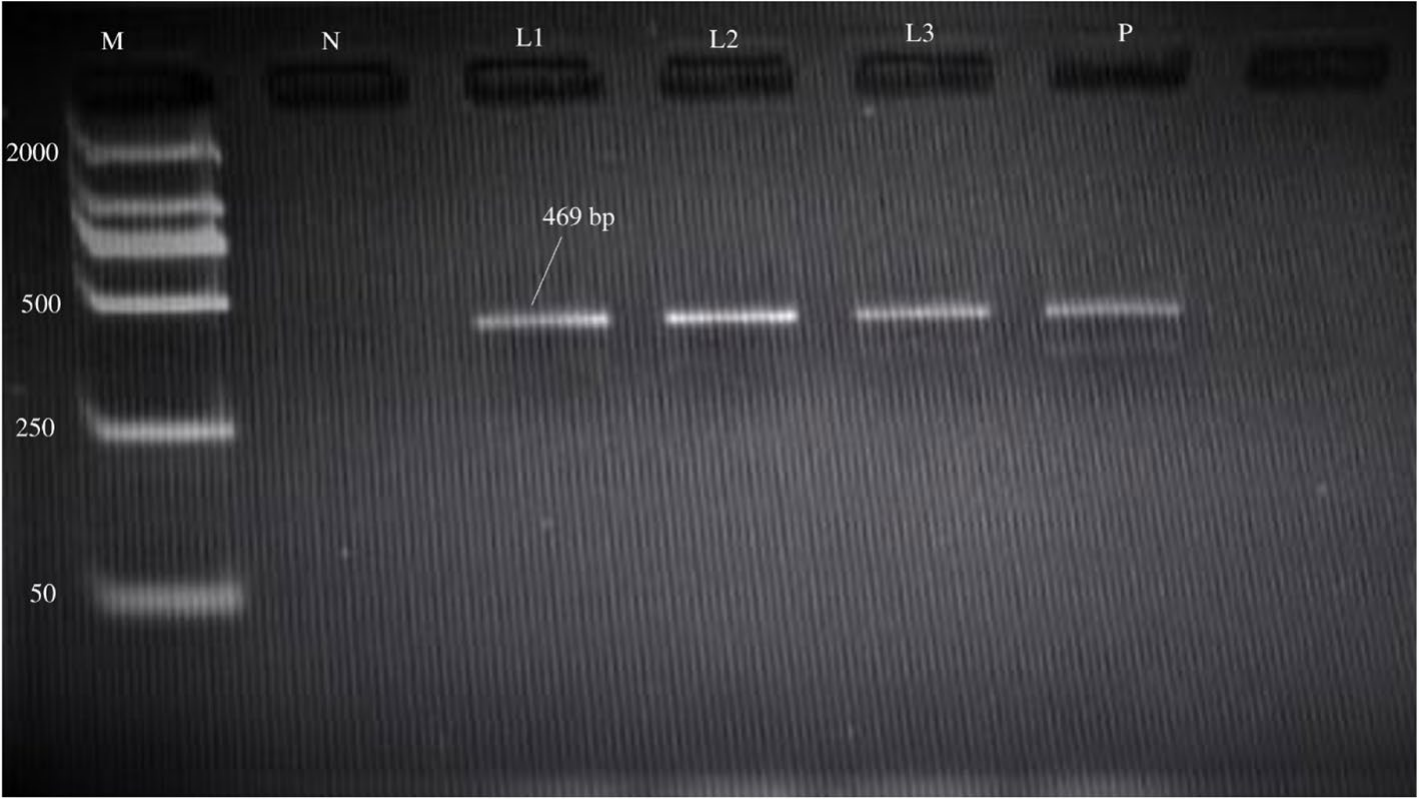
Gel electrophoresis of *T.gondii* B1 gene. M = DNA ladder. N = negative control. P = positive control. L1-L3 represent positive samples of *T. gondii*

DNA was extracted by using DNA extraction kit “Blood Anima Plant DNA Preparation Kit” (Jena Bioscience GmbH, Jena, Germany). Instructions from the manufacturer were followed for DNA extraction. The amplification of the prepared DNA was carried out as described by [8]. For the multiplication of the *T. gondii* B1 gene (469 bp fragment), primers Tg1 (5-AAAAATGTGGGAATGAAAGAG-3) and Tg2 (5-ACGAATCAACGGAACTGTAAT-3) were used. The final volume of each reaction was 25 μl, comprising of 2.4 μl of MgCl_2_ (25 mM), 10X reaction buffer 3.0 μl, dNTPs (500 μM) 1.0 μl, Tg1 (sense primer) 1.0 μl, Tg2 (anti-sense primer) 1.0 μl, Taq DNA polymerase (5 U/μl) 1.0 μl, extracted DNA 5.0 μl and dH_2_O 10.6 μl. The reaction was processed in a thermal cycler (NyxTech, USA) using the program. The initial temperature was 95 °C for 8 min, followed by 35 cycles of 95 °C for 1 min, 52 °C for 30 s, and 72 °C for 1 min, with a final extension temperature of 72 °C for 10 min. Amplified DNA was resolved on a 1.8% agarose gel. A DNA ladder marker of 2000 bp was compared with bands (Fermentas, USA). The bands were resolved by utilizing a gel documentation device under UV lighting as shown in (Fig. 1).

### Statistical analysis

For statistics (SPSS 23.0) software was used to analyze the obtained data by performing different statistical tests, such as a two proportion Z-test for differences between two values, a crosstab test for chi-square, and a one sample nonparametric test for confidence interval to know the differences between different variablesincluding type of cat, gender, region, age, and health conditions. The differences were considered statistically significant if *P* < 0.05 and a 95% confidence interval were taken.

## Results

**Fig. 2:**
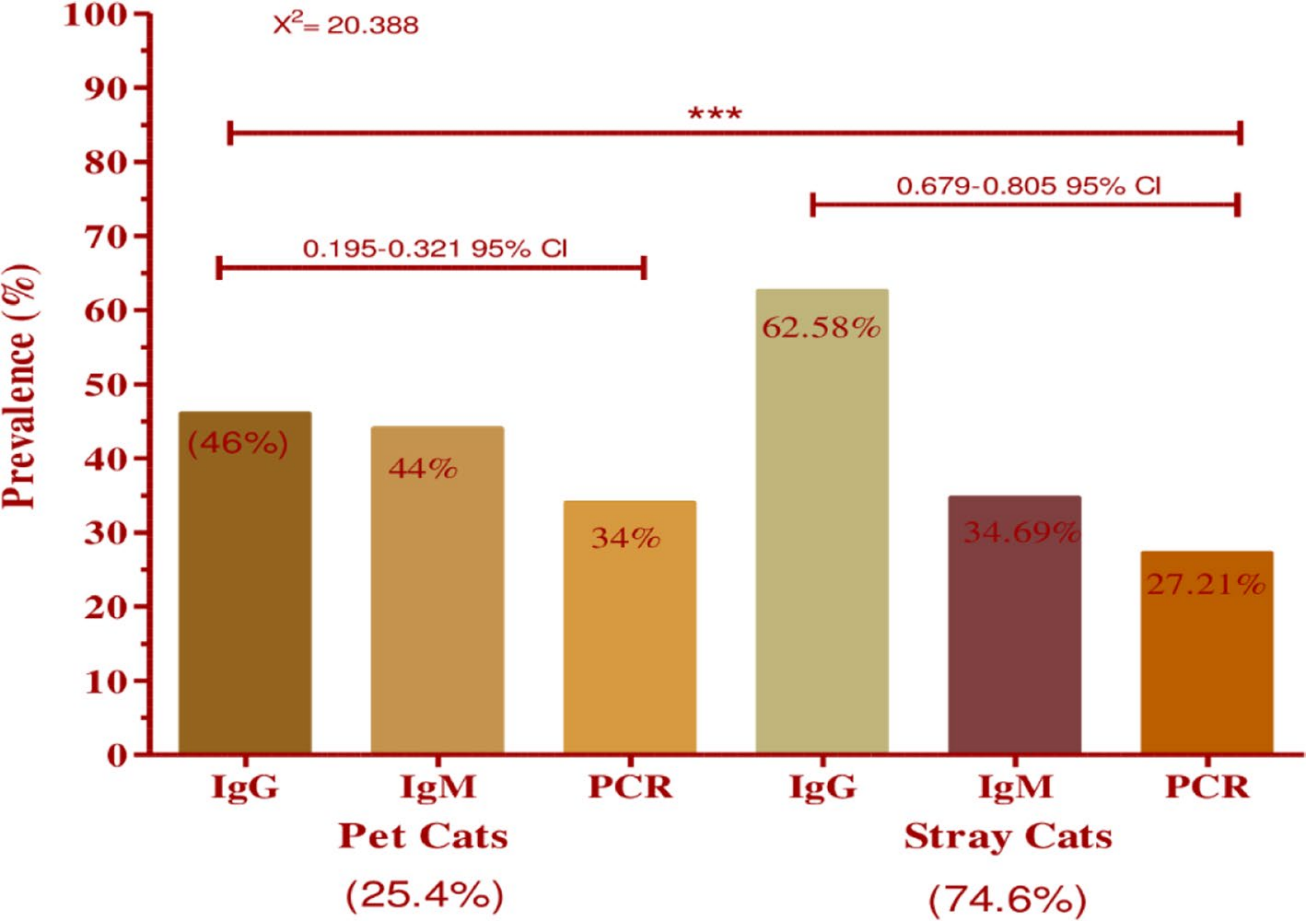
The prevalence rate of *T. gondii* in pet and stray cat in Khyber Pakhtunkhwa, Pakistan. *** showing that *P*-value is (< 0.001) and statistically highly significant, x^2^ is chi-square value and CI is the confidence interval

In the current study, a total of 197 cat samples were analysed through serological (ELISA) and molecular (PCR) techniques in which 152 (77.15%) samples were found positive for toxoplasmosis. Out of 197 samples, 58.37% were recorded as positive against anti-*Toxoplasma* IgG antibodies, 30.45% were found positive against IgM and 23.85% of DNA was successfully amplified through PCR. The study population was divided into stray and pet cats. The present study recorded a higher prevalence of toxoplasmosis in stray cats (74.6%) than in pet cats (25.4%) with highly significant differences (*x*^2^ = 20.388; *p* = 0.0001) as shown in (Fig. 2).

The gender wise prevalence of *T. gondii* was higher in male cats (81.66% as compared to the female cat population of 75.18%). However, the data was statistically non-significant (*x*^2^ = 0.995; *p* = 0.3180). Comparing the prevalence of toxoplasmosis in area, it was found that *T. gondii* was more prevalent (95.55%; 43/45) in district Kohat, followed by district Charsadda (78.57%; 33/42) and Peshawar (78.33%; 47/60) than in Mardan (58%; 29/50) with significant differences (*x*^2^ = 19.149; *p* = 0.0001) as shown in (Table 2).

**Table 2:** Prevalence and Epidemiology of Toxoplasmosis in Stray and Pet Cats of Khyber Pakhtunkhwa, Pakistan

Variable	Diagnostic Test	Overall positive/Total (%)	Test-wise Positive	Prevalence% of tests	95%CI	x^**2**^	***P***-Value
**Gender**							
Male	IgG	49/60(81.66)	37	61.66	0.241–0.374	0.995	0.3180
	IgM		21	35			
	PCR		19	31.66			
Female	IgG	103/137(75.18)	78	56.93	0.626–0.759		
	IgM		39	28.46			
	PCR		28	20.43			
**Regions**							
Peshawar	IgG	47/60(78.33)	37	54.06	0.241–0.374	19.149	0.0001
	IgM		16	24.24			
	PCR		14	21.21			
Charsadda	IgG	33/42(78.57)	23	54.76	0.158–0.277		0.0001
	IgM		14	33.33			
	PCR		10	23.8			
Mardan	IgG	29/50(58)	27	54	.195–.321		0.0001
	IgM		11	22			
	PCR		7	14			
Kohat	IgG	43/45(95.55)	28	62.22	0.172–0.293		0.0001
	IgM		19	42.22			
	PCR		16	35.55			
**Age**							
≤ 2 Years	IgG	37/61(60.65)	24	39.34	0.246–0.379	15.778	0.0001
	IgM		16	26.22			
	PCR		13	21.31			
2–4 Years	IgG	71/88(80.68)	56	63.63	0.376–0.519		
	IgM		25	28.4			
	PCR		19	21.59			
≥ 4 Years	IgG	44/48(91.66)	35	72.91	0.185–0.310		
	IgM		19	39.58			
	PCR		15	31.25			
**Health**							
Healthy	IgG	102/138(73.91)	81	58.69	0.631–0.764	3.293	0.1930
	IgM		27	19.56			
	PCR		17	12.31			
Poor	IgG	38/46(82.6)	24	52.17	0.176–0.299		
	IgM		22	47.82			
	PCR		19	41.3			
Bad	IgG	12/13(92.3)	10	76.92	0.036–0.110		
	IgM		11	84.61			
	PCR		11	84.61			

The age-wise prevalence of toxoplasmosis reveals a gradual rise in infection in different age groups. Toxoplasmosis was detected highest in the higher age group, i.e. age equal to 4 years or above, (91.66%) as they were highly vulnerable to infection, while cats younger or equivalent to 2 years of age were least vulnerable to toxoplasmosis (60.65%). The differences were highly significant (*x*^2^ = 15.778; *p* = 0.0001). Depending upon the health conditions of the cats, toxoplasmosis was found to be highest in poor health conditions, at (92.3%) while the lowest occurrence was in healthy conditions, at (73.91%) with non-significant differences ((*x*^2^ = 3.293; *p* = 0.1930) as seen in (Table 2).

Based on infection types in the current study, all results were categorized as acute, chronic and reactivated. Cases were categorized as chronic when only IgG antibodies were recorded as positive for toxoplasmosis. The presence of only IgM antibodies or IgM antibodies along with PCR positive was considered as an acute infection. In such cases, where the subjects were recorded positive for IgG + IgM or positive through PCR + IgM + IgG were considered as reactivated infections (Table 3).

**Table 3:** Overall Picture of Cats Toxoplasmosis in studied Population

Infection Type	IgG (p)	IgM (p)	PCR (p)	Examined	Positive	Prevalence%
**Chronic**	P	N	N	197	92	46.7
**Acute**	N	P	N	197	6	3.04
	N	P	P	197	31	15.73
**Reactivated** (Chronic)	P	P	N	197	7	3.55
	P	P	P	197	16	8.12
**Negative**	N	N	N	197	45	22.84

P is denoted for positive and N for negative samples of *T. gondii*

## Discussion

*Toxoplasma gondii* has a zoonotic ability to spread globally. It is readily transmissible between man and the host vertebrate reservoir. Keeping in view the zoonotic importance of *T. gondii*, the present study has been conducted. Studies carried out around the world display a 2.7 to 90% occurrence of cat toxoplasmosis in different environmental places, climate, socioeconomic conditions, history, and variations in different testing techniques [10]. The overall seroprevalence of *T. gondii* in stray and pet cats reported in the present study was 77.15% with indirect ELISA and PCR techniques. In contrast, a survey conducted in Faisalabad and Lahore (Pakistan) reported 60 and 56% occurrence of toxoplasmosis respectively by different techniques [11, 12], which was slightly different from our findings. The reason behind the dissimilarity may be the use of different methods, origin and size of studied samples. As in the current study, we have used more specific, modern, reliable, and sensitive techniques such as indirect ELISA and PCR.

Present study documents a higher occurrence of IgG antibodies at 58.37% as compared to IgM antibodies at 30.45% with the indirect ELISA method. This might be due to IgG antibodies being initially absent during primary infection, but after some weeks they rise to protective levels and remain detectable for years, while the lower occurrence of IgM antibodies is that they increase within days, and usually decrease in a couple of weeks. However, in some cats with chronic infection, IgM persists [13]. IgM does not always show a recent infection, but the results of a combination of different antibodies and molecular investigations would be valuable in confirming a chronically reactivated stage; recirculation of the parasite in the blood and serological testing suggest a prior immune response [8]. It is predicted that there will be a high number of chronic cases while studying the cat population. Cats seropositive for IgG are generally known as chronically infected [14]. Cases in which cats were positive for IgG but with elevated IgM levels are an indication of revival. However, it also indicates that some weeks after infection, they may switch from IgM to IgG [15]. Cats found positive for IgG + IgM and IgG + IgM + PCR are proposed to be named as chronic reactivated cases. Reactivation of chronically compromised stages includes cyst reactivation, conversion of bradyzoites to tachyzoites, and does not usually require a new entero-epithelial process or oocyst excretion [6]. The presence of acute cases suggests ongoing disease activity and a significant probability of protozoan interaction with cats. The risk of cats coming in contact with infected cysts while hunting infected animals or eating supplied food may influence the incidence of acute cases, showing the importance of providing cats with an adequate diet [16]. As a definite host of *T. gondii*, cats play a key role in parasite transmission and regular contact with cats usually raises the risk of infection for both cats and humans, as children play outside in the dirt and pet cat owners have direct contact [17, 18].

Epidemiological surveys carried out in several countries reported higher occurrence of toxoplasmosis in stray cats, with a considerably lower prevalence rate in pet cats [10]. The current study also shows highly significant seroprevalence of *T. gondii* in stray cats 74.6% in contrast to pet cats 25.4%. Current study reports higher seroprevalence of 81.66% in males as compared to female cats, 75.81%, but the data was statistically non-significant. Many studies acclaim higher incidence in males related to their territorial habits, since they have a wide variety of areas for activity than those of females [19]. Current findings agree with the study conducted in China, where the male population was documented with a higher prevalence rate of 22.16% than female felines at 20.14%, with a non-significant difference [20]. However, findings of the current study oppose the results from Faisalabad (Pakistan), where the infection rate was lower in males at 40% as compared to females at 70% [12]. The present study consisted of cats from healthy to poor and bad health conditions. Cats with bad and poor body conditions were most susceptible to *T. gondii* infection, 92.3 and 82.6% respectively, in comparison to cats with healthy conditions, 73.91%. Due to poor diet, cats remain unhealthy and damage their immune systems afterwards. Similarly, prolonged illness and infection levels in the blood and gut make cats prone to multiple diseases [8, 21]. Current study reveals that gradual rise in prevalence rate with age is statistically highly significant, while lower most in cats having age less than 2 years 60.65% followed by those aging between 2 and 4 years 80.68% and highest in felines above 4 years old 91.66%, possibly due to adult felines have greater exposure to *T. gondii* in the course of their life. These findings are consistent with what are widely stated globally in the literature [10].

This research was conducted in four distinct districts of Khyber Pakhtunkhwa, i.e. Peshawar, Charsadda, Mardan, and Kohat. The prevalence of *T. gondii* was observed higher in district Kohat (95.55%), statistically highly significant *P* < 0.001, which might be due to the mountainous region having an average warm temperature. The district mainly comprises of rural areas with a low urban population, as cats have outdoor access to food in rural areas, depending solely on wild birds and rodents living in this area, which play an important role in toxoplasma epidemiology [8]. Low rates of infection were observed in the districts of Charsadda (78.57%), Peshawar (78.33%) and Mardan (58%) as compared to Kohat. Lower infection rates might be due to feline feeding habits and better grooming in the region, as these are heavily populated (urban rather than rural) districts of the province. Also, cats do not have outdoor access to wild birds and mice for hunting, which play an important part in the transmission of the disease. Normally, the feline populations in these areas typically feed on well prepared and hygienic commercial food, which minimizes the transmission of contamination to the community.

## Conclusion

The outcomes of the current study revealed that *T. gondii* infection is extensively spread in the studied stray and pet cat’s population and is a serious threat to environmental contamination. This means that the human population is at a higher risk of obtaining the infection. Therefore, this study commends more serological and molecular diagnosis of acute and chronic toxoplasmosis in the future, as well as genotyping of *T. gondii* strains from different hosts to forecast the current approach for prevention and control of this zoonotic parasite.

## Data Availability

The datasets used and/or analysed during the current study are available from the corresponding author on reasonable request.

## Abbreviations

dNTPs: Dinucleotides triphosphate
SPSS: Statistical package for social sciences
EDTA: Ethylene Diamine Tetra Acetic Acid
ELISA: Enzyme-linked immunosorbent assay
LAT: Latex agglutination test
Mgcl_2_: Magnesium Chloride
